# Osteoarthritis Development Following Meniscectomy vs. Meniscal Repair for Posterior Medial Meniscus Injuries: A Systematic Review

**DOI:** 10.3390/medicina60040569

**Published:** 2024-03-30

**Authors:** Mihai Hurmuz, Mihai Ionac, Bogdan Hogea, Catalin Adrian Miu, Fabian Tatu

**Affiliations:** 1 Doctoral School, “Victor Babes” University of Medicine and Pharmacy Timisoara, Eftimie Murgu Square 2, 300041 Timisoara, Romania; hurmuz.mihai@umft.ro; 2 Department XV, Discipline of Orthopedics, “Victor Babes” University of Medicine and Pharmacy Timisoara, Eftimie Murgu Square 2, 300041 Timisoara, Romania; miu.catalin@umft.ro (C.A.M.); tatu.fabian@umft.ro (F.T.); 3 Orthopedics Unit, “Victor Popescu” Emergency Military Hospital, Gheorghe Lazar Street 2, 300080 Timisoara, Romania; 4 Department X, Discipline of Vascular Surgery, “Victor Babes” University of Medicine and Pharmacy Timisoara, Eftimie Murgu Square 2, 300041 Timisoara, Romania; mihai.ionac@umft.ro; 5 Profesor Universitar Doctor Teodor Șora Research Centre, “Victor Babes” University of Medicine and Pharmacy Timisoara, Eftimie Murgu Square 2, 300041 Timisoara, Romania

**Keywords:** meniscal injuries, meniscectomy, meniscal repair, osteoarthritis, knee, posterior horn meniscus

## Abstract

This systematic review aims to evaluate critically and synthesize the existing literature on the outcomes of meniscectomy versus meniscal repair for posterior medial meniscus injuries, with a focus on osteoarthritis (OA) development. We sought to assess the incidence of OA following both treatment modalities, compare functional outcomes post-treatment, and identify factors influencing treatment choice, providing evidence-based recommendations for clinical decision-making. A comprehensive search strategy was employed across PubMed, Scopus, and Embase up until December 2023, adhering to PRISMA guidelines. The primary outcomes included OA development, functional knee outcomes, and quality of life measures. Six studies met the inclusion criteria, encompassing 298 patients. The systematic review revealed a significant association between meniscal repair and decreased progression of OA compared to meniscectomy. Meniscectomy patients demonstrated a 51.42% progression rate towards OA, significantly higher than the 21.28% observed in meniscal repair patients. Functional outcomes, as measured by the International Knee Documentation Committee (IKDC) and Lysholm scores, were notably better in the repair group, with average scores of 74.68 (IKDC) and 83.78 (Lysholm) compared to 67.55 (IKDC) and 74.56 (Lysholm) in the meniscectomy group. Furthermore, the rate of complete healing in the repair group was reported at 71.4%, as one study reported, indicating a favorable prognosis for meniscal preservation. However, these pooled data should be interpreted with consideration to the heterogeneity of the analyzed studies. Meniscal repair for posterior medial meniscus injuries is superior to meniscectomy in preventing OA development and achieving better functional outcomes and quality of life post-treatment. These findings strongly suggest the adoption of meniscal repair as the preferred treatment modality for such injuries, emphasizing the need for a paradigm shift in clinical practice towards preserving meniscal integrity to optimize patient outcomes.

## 1. Introduction

The meniscus, a key fibrocartilaginous structure within the knee joint, consists of the medial and lateral menisci [1,2,3,4,5,6,7,8,9,10]. These crescent-shaped discs act as shock absorbers, distribute load, and enhance joint stability. Integral to knee function, the menisci mitigate the forces transmitted across the knee, reducing the risk of cartilage damage and osteoarthritis [11,12,13,14,15]. The medial meniscus, being C-shaped and firmly attached to the knee’s capsule, is particularly vulnerable to injury, underscoring the importance of preserving its integrity for maintaining knee health [16,17,18,19,20].

The posterior root of the medial meniscus plays a critical role in maintaining knee joint homeostasis and biomechanical integrity [21,22,23,24]. Meniscal injury can significantly impact the knee biomechanics, while its healing process can be influenced by various comorbidities, surgical procedures, or infections [25,26,27,28,29,30,31]. Its injury not only predisposes the knee to altered load distribution but also accelerates the process of degenerative changes, leading to osteoarthritis (OA) [32]. This phenomenon has been increasingly recognized in orthopedic research, given the prevalence of knee injuries across various populations [33]. Studies have shown that the integrity of the medial meniscus, particularly its posterior root, is essential in preserving knee joint function and delaying the onset of OA. The degradation of this structure significantly impacts knee biomechanics, increasing the risk for the development of OA [34].

Meniscal injuries are among the most common knee injuries encountered in clinical practice, with the posterior horn of the medial meniscus being particularly susceptible due to its anatomical and functional characteristics [35,36,37]. The management of these injuries remains a subject of considerable debate, with options ranging from conservative management to surgical interventions such as meniscectomy and meniscal repair [38,39]. Historically, meniscectomy was the standard treatment for meniscal tears, including those at the posterior root [40]. However, this approach has been linked to unfavorable long-term outcomes, including the accelerated development of OA [41,42]. The recognition of these adverse outcomes has shifted the treatment paradigm towards preserving meniscal tissue through meniscal repair techniques [43].

Recent advancements in arthroscopic surgery have made meniscal repair a more feasible and attractive option for the management of posterior medial meniscus injuries [44]. Comparative studies between meniscectomy and meniscal repair have provided valuable insights into their respective impacts on knee joint health, suggesting that patients undergoing meniscal repair exhibit better long-term outcomes regarding knee function and a lower incidence of OA compared to those who undergo meniscectomy [45], although one study found that meniscal repair was detrimental to meniscectomy from baseline to 52 weeks, and long-term at five years post-surgery [46].

Despite these advancements, the decision-making process regarding the optimal management strategy for medial meniscus injuries remains complex. Factors such as the type of meniscal tear, patient age, activity level, and presence of concurrent knee pathologies play an important role in determining the most appropriate treatment approach [47]. Therefore, the aim of this systematic review was to critically evaluate and synthesize the existing literature on the outcomes of meniscectomy versus meniscal repair for injuries at the posterior root of the medial meniscus, with a particular focus on the development of osteoarthritis. Objectives included assessing the incidence of OA following both treatment modalities, comparing functional outcomes and quality of life post-treatment, and identifying factors influencing the choice of treatment. This review sought to provide evidence-based recommendations to inform clinical decision-making and improve patient outcomes in the management of posterior medial meniscus injuries. 

## 2. Materials and Methods

### 2.1. Protocol and Registration

This systematic review was carried out in December 2023 and employed a search strategy deployed across three databases (PubMed, Scopus, and Embase). The search strategy utilized an extensive array of keywords and phrases intricately linked to the study’s objectives, specifically targeting the meniscal injuries and their subsequent management options, as well as their implications for osteoarthritis development. The list of key search terms included: “posterior medial meniscus injuries”, “posterior horn of the medial meniscus”, “posterior root tear of the medial meniscus”, “meniscectomy”, “meniscal repair”, “osteoarthritis development”, “knee surgery outcomes”, “arthroscopic surgery”, “knee joint health”, “biomechanics of the knee”, “degenerative joint disease”, “surgical intervention efficacy”, “meniscal preservation”, “load distribution in the knee”, “joint space narrowing”, and “cartilage health.”

To construct an effective search query, Boolean operators (AND, OR, NOT) were adeptly used to combine these terms in a manner that refined and focused the search. The search string was designed as follows: (((“posterior medial meniscus injuries” OR “posterior horn of the medial meniscus” OR “medial meniscus” OR “meniscus tear” OR “meniscus injury” OR “meniscus lesions” OR “posterior root tear of the medial meniscus”) AND (“meniscectomy” OR “meniscal repair”)) AND (“osteoarthritis development” OR “knee osteoarthritis”) AND (“surgical outcomes” OR “long-term outcomes” OR “treatment efficacy”) AND (“knee joint health” OR “biomechanics of the knee” OR “degenerative joint disease” OR “meniscal preservation” OR “load distribution in the knee” OR “joint space narrowing” OR “cartilage health”)).

This protocol, structured in accordance with the Preferred Reporting Items for Systematic Reviews and Meta-Analyses (PRISMA) guidelines, was meticulously designed to guarantee a systematic, transparent, and replicable methodology [48]. To further the transparency and accessibility of our research process and findings, this review was registered with the Open Science Framework (OSF) with the registration number osf.io/43hwg.

### 2.2. Eligibility Criteria and Definitions

The selection of studies was governed by a set of defined inclusion and exclusion criteria. Inclusion criteria comprised the following: (1) Studies must involve patients diagnosed with injuries to the posterior root of the medial meniscus, regardless of age, gender, and activity level; (2) Included studies should compare outcomes of meniscectomy and meniscal repair interventions specifically for the posterior medial meniscus injuries; (3) Studies must report on the development of osteoarthritis as a primary or secondary outcome, assessed through clinical evaluation, imaging studies (MRI or X-ray), or symptomatic assessment. Additional outcomes of interest include functional knee outcomes, quality of life measures, and any reported complications or re-interventions; (4) A broad range of study designs will be considered, including randomized controlled trials, cohort studies, case-control studies, cross-sectional studies, and case series.

Exclusion criteria comprised the following: (1) Non-human studies—studies involving in vitro or animal models will be excluded to focus exclusively on outcomes relevant to human patients; (2) Irrelevant populations—studies not specifically addressing injuries to the posterior root/horn of the medial meniscus or their management via meniscectomy or meniscal repair will be excluded; (3) Non-specific outcomes—studies failing to clearly report on the development of osteoarthritis or lacking specific outcome measures related to knee function, quality of life, or long-term complications post-intervention will be omitted; (4) Insufficient data—studies lacking sufficient detail to allow a comprehensive understanding of the methodologies used, results obtained, and conclusions drawn will be excluded to maintain the review’s credibility and reliability; (5) Grey literature—to ensure the inclusion of high-quality, peer-reviewed evidence, grey literature such as non-peer-reviewed articles, case reports, proceedings, conference abstracts, general reviews, commentaries, and editorials will be excluded; (6) Healthy young individuals and sports injuries will be excluded.

### 2.3. Definitions

Osteoarthritis, in the context of this review, refers to the degenerative joint disease following the criteria established by the American College of Rheumatology (ACR) and is corroborated by radiographic assessment using the Kellgren–Lawrence grading system [49]. Medial meniscus tears of the posterior horn are classified based on their morphology (e.g., radial, horizontal, complex, and root tears) and are diagnosed through magnetic resonance imaging (MRI) findings, supplemented by clinical examination. Meniscectomy involves the partial or total removal of the damaged meniscal tissue. It is indicated for tears not amenable to repair or in cases where repair is unlikely to succeed due to poor tissue quality or avascularity. Meniscectomy aims to alleviate symptoms by removing the unstable meniscal fragments that could cause mechanical symptoms, yet it is known to increase the risk of developing OA due to the loss of meniscal function in load distribution and shock absorption. Meniscal repair aims to preserve meniscal tissue and restore its normal anatomy and function. Meniscal repair is preferable for tears with a high potential for healing, particularly in the “red–red” or “red–white” zones with adequate blood supply. Techniques vary from all-inside, inside-out, to outside-in suture repairs, depending on the tear location and surgeon preference, aiming to maintain meniscal integrity, thereby preserving knee biomechanics and reducing the risk of OA [50].

### 2.4. Data Collection Process

**Figure 1:**
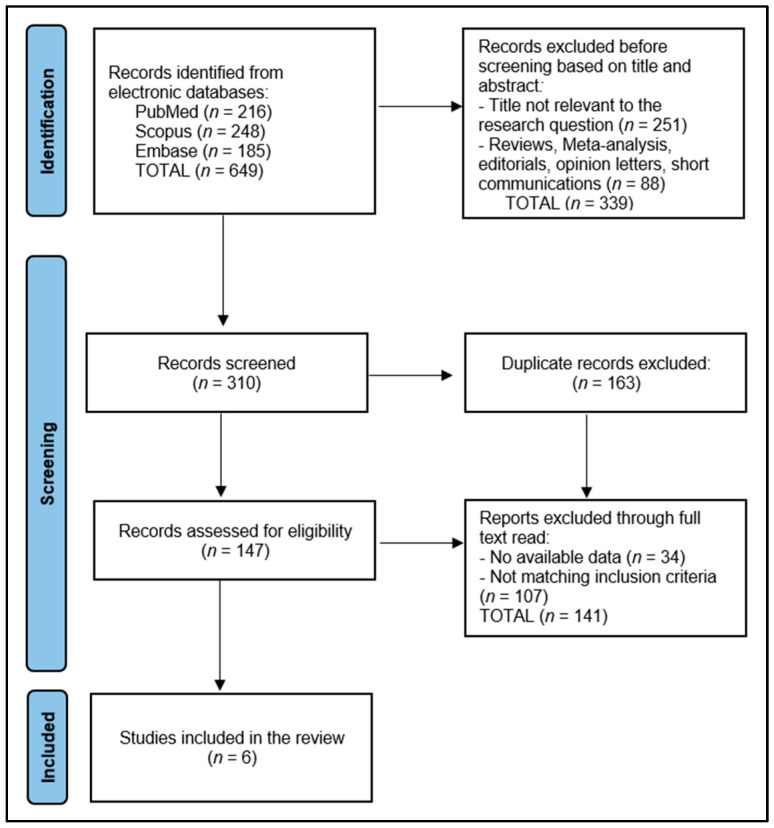
PRISMA Flow Diagram.

The search across PubMed, Scopus, and Embase yielded 649 articles. After removing 339 publications before screening based on title and abstract, 310 articles remained for preliminary screening. Two independent reviewers screened titles and abstracts based on inclusion and exclusion criteria related to posterior medial meniscus injuries, interventions, and osteoarthritis development, resolving discrepancies through discussion or consultation with a third reviewer if needed. This initial screening excluded 163 duplicates, with 147 publications remaining for eligibility assessment, to be evaluated for their relevance and data quality, focusing on study design, population, interventions, and outcomes regarding osteoarthritis development and knee function. Finally, a total of 6 studies clearly provided insights into the effects of meniscectomy versus meniscal repair on osteoarthritis development in patients with posterior medial meniscus injuries, as presented in Figure 1.

### 2.5. Risk of Bias and Quality Assessment

For assessing study quality and bias risk, our review applied a dual method, blending qualitative and quantitative analyses. Observational study quality was assessed using the Newcastle–Ottawa Scale, focusing on group selection, group comparability, and outcome or exposure assessment. Studies received a cumulative star score, categorizing their quality as low, medium, or high, enabling precise quality assessments. Two independent researchers evaluated each study, with any disagreements resolved via discussion or a third reviewer’s input.

## 3. Results

### 3.1. Study Characteristics

The systematic review encompassed in the final analysis a total of six studies [51,52,53,54,55,56], as delineated in Table 1, conducted over a span from 2011 to 2022. These investigations, originating from a diverse range of countries including South Korea, the United States, Spain, and China; all adopted a retrospective cohort design, with the exception of one case-control study by Dzidzishvili et al. [55] from Spain in 2022. All of these studies were classified as having medium quality of evidence, signifying a moderate level of confidence in the reliability of their findings.

The utilization of retrospective cohort designs in five out of the six studies, specifically Kim et al. [51], Chung et al. [52], Bernard et al. [53], Kim et al. [54], and Su et al. [56], highlights a prevalent approach within this research area, focusing on the examination of historical data to infer outcomes related to osteoarthritis development post-surgery. The singular case-control study by Dzidzishvili et al. [55] offered a comparative analysis. Moreover, the geographical diversity, with studies conducted in South Korea [51,52,54], the United States [53], Spain [55], and China [56], enriches the review’s global perspective on the treatment for posterior medial meniscus injuries. However, the concentration of studies from South Korea, accounting for half of the included research, may indicate a regional interest or expertise in this domain of orthopedic surgery. 

### 3.2. Patients’ Characteristics

Data presented in Table 2 comprise a total of 298 participants, with a total of 144 patients from the meniscectomy group and 154 from the repair group. A precise delineation of age revealed an average of 55.13 years for the meniscectomy group and 54.19 years for the repair group, suggesting a relatively middle-aged cohort undergoing these orthopedic interventions.

**Figure 2:**
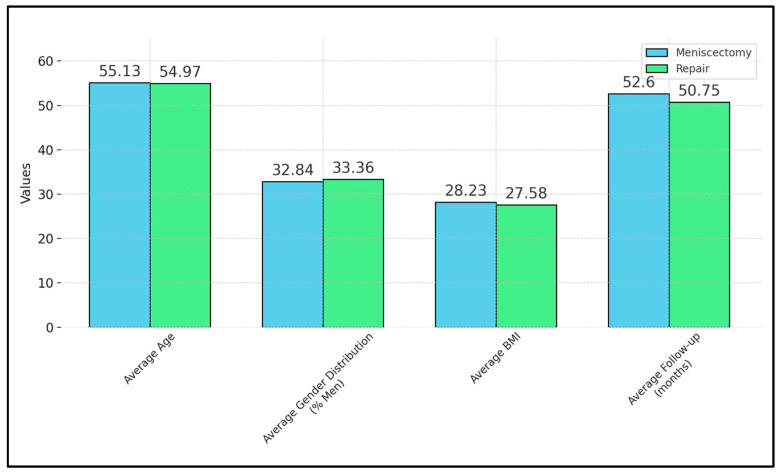
Patient characteristics.

The gender distribution demonstrated a slight male predominance, with 32.48% in the meniscectomy group and a marginally higher 33.36% in the repair group, underscoring the gender dynamics within these surgical interventions. Moreover, the average BMI was noted at 28.23 in the meniscectomy cohort and slightly lower at 27.58 in the repair group, indicating a broadly comparable baseline nutritional status across both surgical groups. Furthermore, the follow-up period averaged 52.60 months for the meniscectomy group and was slightly less, at 50.75 months, for the repair group, as described in Figure 2.

### 3.3. Disease Characteristics

Table 3 provides detailed insights into preoperative condition severity, measured through Kellgren–Lawrence grades, International Knee Documentation Committee (IKDC) scores, Lysholm scores, and other findings including joint space measurements. Kellgren–Lawrence grading revealed variability across studies with respect to osteoarthritis severity prior to surgery. Notably, Kim et al. [51] reported 33.3% of meniscectomy cases and 25% of the repair cases were Grade 3–4, indicating advanced osteoarthritis. Conversely, Chung et al. [52] and Kim et al. [54] observed no patients within these high-grade categories in either treatment group, suggesting less severe osteoarthritic conditions at the outset. Dzidzishvili et al. [55] found a higher prevalence of Grade 3–4 osteoarthritis in the meniscectomy group (34.3%) compared to the repair group (20.0%), while Bernard et al. [53] and Su et al. [56] utilized median scores, revealing a slight variance in osteoarthritis severity between treatment groups but generally indicating mild to moderate conditions.

The IKDC scores, which were reported in four studies [51,52,54,56], averaged 37.2 for meniscectomy and 39.1 for repair groups, pointing towards a slightly better knee function in the repair group preoperatively. However, Bernard et al. [53] and Dzidzishvili et al. [55] did not report these scores, limiting a comprehensive cross-study comparison.

Similarly, Lysholm scores were only partially reported. Where available, they reflected modestly better preoperative function or symptoms in the repair group, with averages of 49.9 for meniscectomy and 50.0 for repair across the studies reporting this outcome [51,52,54,56]. These scores suggest that, on average, patients undergoing repair might have been in slightly better condition or experienced less severe symptoms before surgery.

Joint space measurements further underscored the nuanced differences between groups. Kim et al. [51], Chung et al. [52], and Kim et al. [54] presented measurements indicating a generally similar preoperative joint space in meniscectomy and repair groups. The smallest reported differences were in the study by Su et al. [56], with a 3.4 mm joint space in meniscectomy cases versus 3.2 mm in repair cases, suggesting minimal discrepancy in joint degradation. Other findings varied across studies, with the Tegner score reported by Chung et al. [52] at 2.7 for both groups, indicating a similar activity level. Dzidzishvili et al. [55] reported higher rates of cartilage degeneration in the meniscectomy group through Outerbridge and LaPrade grading, highlighting the potential for more severe intra-articular damage in these patients.

### 3.4. Postoperative Outcomes

**Figure 3:**
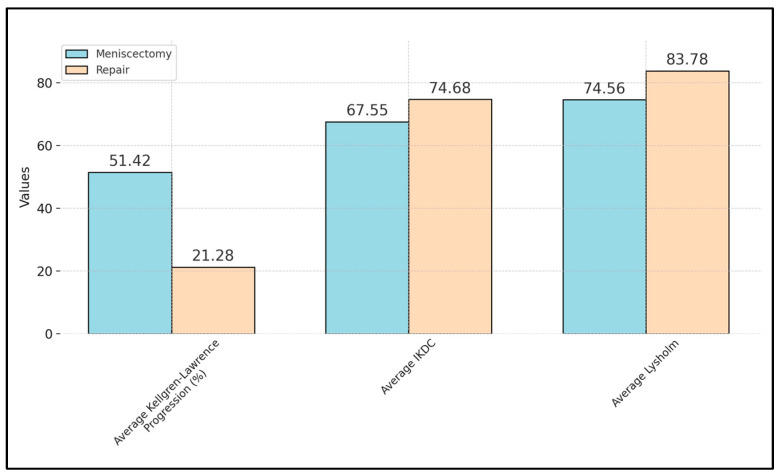
Aggregate mean values of patient outcomes after meniscectomy and meniscal repair.

Collectively, the studies revealed a clear trend in Kellgren–Lawrence progression, with meniscectomy groups exhibiting significantly higher rates of osteoarthritis development compared to their meniscal repair counterparts, as described in Table 4. For instance, the progression rates reported by Kim et al. [51] and Chung et al. [52] were markedly higher in the meniscectomy groups (75.0% and 85.0%, respectively) than in the repair groups (30.0% and 20.0%, respectively). The aggregate mean of Kellgren–Lawrence progression rate across all six studies was observed in 51.42% of patients after meniscectomy, compared with only 21.28% in the meniscal repair group (Figure 3), indicating a pronounced protective effect of meniscal repair against the progression of osteoarthritis.

When examining functional outcomes through IKDC and Lysholm scores, an average IKDC score of 71.3 for meniscectomy and 73.7 for repair groups, alongside an average Lysholm score of 82.5 for meniscectomy and 86.6 for repair groups, were observed. The aggregate average IKDC score across all studies was 67.55 among patients after meniscectomy, compared with 74.68 in the meniscal repair group. These averages underscore the superior functional recovery in patients undergoing meniscal repair, suggesting not only a preservation of knee integrity but also an enhanced quality of life post-surgery.

The average rate of complete healing in the repair group, as evidenced by the study of Su et al. [56] showing a 71.4% healing rate, further solidifies the argument in favor of meniscal repair. This is contrasted against the backdrop of higher osteoarthritis progression and the necessity for total knee replacement (TKR) in the meniscectomy groups, where Kim et al. [51] reported a 10.7% rate of progression to TKR in the meniscectomy group compared to a 0.0% rate in the repair group. The aggregate mean Lysholm score across all studies was 74.56 in the meniscectomy group vs. 83.78 in the meniscal repair group, as presented in Figure 3.

## 4. Discussion

### 4.1. Summary of Evidence

Across the analyzed studies, a clear distinction in postoperative outcomes emerges, underlining the critical importance of surgical choice on the progression of osteoarthritis and overall knee function recovery after meniscectomy or meniscal repair. Notably, the aggregate data reveal that meniscal repair consistently leads to superior outcomes in terms of mitigating osteoarthritis development and enhancing functional scores after posterior root lesions of the medial meniscus, as evidenced by IKDC and Lysholm metrics.

The findings, particularly those concerning Kellgren–Lawrence progression rates, highlight a significant differential impact of surgical options on osteoarthritis development. With meniscectomy groups showing a notably higher progression rate towards osteoarthritis compared to repair groups, the data suggest a compelling argument for the adoption of meniscal repair as a strategy to preserve knee integrity. This distinction not only reflects the inherent benefits of meniscal repair in maintaining joint health, as previously suggested [57,58,59], but also suggests potential long-term advantages, including a reduced need for subsequent surgical interventions such as total knee replacement.

The clinical implications of these findings are profound, especially when considering the preoperative condition severity and baseline patient characteristics. Despite the relatively similar demographic and clinical baseline profiles across both groups, the outcomes starkly diverge post-surgery, emphasizing the role of surgical technique in determining patient trajectory. This aspect is particularly critical given the middle-aged demographic of the cohort, for whom quality of life and functional capacity post-surgery are paramount concerns [60,61,62,63,64,65,66,67,68,69,70,71,72].

Furthermore, the nuanced analysis of functional outcomes through IKDC and Lysholm scores across the studies underscores the tangible benefits of meniscal repair in terms of knee function and symptom alleviation. The slight, yet consistent, superiority in scores for the repair group points to a better recovery trajectory, which, when coupled with lower rates of osteoarthritis progression, positions meniscal repair as the preferable surgical approach for patients with posterior medial meniscus injuries.

The integration of these findings into clinical practice demands a re-evaluation of current treatment paradigms. With meniscal repair demonstrating not only a good safety profile but also a clear advantage in preserving knee joint function and integrity, the evidence calls for a more discerning approach to surgical decision-making. This shift towards meniscal repair, supported by robust clinical outcomes, advocates for a patient-centered approach that prioritizes long-term joint health and functional recovery, potentially redefining standard care practices for individuals with meniscal injuries.

In light of our study’s findings, the research conducted by Dzidzishvili et al. provides compelling histopathologic insights into the treatment of meniscus root tears and its implications for osteoarthritic development, although in rabbit models, instead of humans [73]. The authors delineated the osteoarthritic changes across three experimental groups—partial meniscectomy, conservative treatment, and meniscus root repair—revealing that the repair group exhibited the least severe cartilage damage with a mean score of 2.5, compared to more pronounced OA signs in the meniscectomy group (mean score of 16) and the CT group (mean score of 5). This gradation of osteoarthritic severity, particularly the pronounced difference between the repair and meniscectomy groups, closely mirrors the trends observed in our systematic review. Our analysis similarly highlighted a reduced progression of OA and better functional outcomes in patients undergoing meniscal repair versus those subjected to meniscectomy. Dzidzishvili et al. findings—showcasing significantly less severe degenerative changes post-repair—lend a quantitative basis to the argument for meniscal repair as a superior surgical strategy. This congruence between Dzidzishvili et al. laboratory data and our clinical evidence underlines the critical role of surgical intervention in moderating OA trajectory, advocating for meniscus root repair as a preferential treatment to forestall the histopathologic advance of knee osteoarthritis.

The systematic review by Wang et al. [74] delved into the effectiveness of medial meniscal posterior root tear repair, both with and without the addition of high tibial osteotomy (HTO), in addressing knee joint lesions. This comprehensive analysis, which encompassed fifteen studies featuring 625 cases, revealed that repair surgeries significantly improved clinical outcomes, particularly noted in groups undergoing repair alone. Despite these advancements, both strategies—repair alone and in conjunction with HTO—showed a similar degree of osteoarthritis progression over approximately a two-year follow-up period, as evidenced by radiologic outcomes. This finding underscores a critical point: while HTO can be a valuable adjunct in treating patients with severe osteoarthritis and varus alignment, the decision between opting for repair alone or in combination with HTO remains a subject of debate. The conclusion of Wang et al. suggests a tailored approach, considering the Kellgren–Lawrence grade, to optimize patient prognosis, highlighting the need for further large-scale, randomized control studies to refine clinical decision-making in the management of medial meniscal posterior root tears.

The study by Bottomley et al. [75] presents a compelling argument in favor of meniscal repair over arthroscopic meniscectomy, a conclusion that resonates with the findings of our systematic review. By evaluating clinical outcomes through a myriad of patient-reported outcome measures, Bottomley et al. not only highlight the significant clinical benefit of surgical intervention in patients with isolated meniscal tears but also emphasize the superior efficacy of meniscal repair in enhancing patient outcomes compared to meniscectomy. This distinction, underscored by a rigorous comparison that revealed meniscal repair as yielding notably better clinical outcomes at a medium-term follow-up, provides a critical insight into the debate surrounding the optimal surgical treatment for meniscal tears. Aligning with our systematic review’s aggregated evidence, The Bottomley et al. study further bolsters the argument for a paradigm shift towards meniscal preservation strategies in orthopedic surgery. It underscores the importance of considering long-term knee health and functionality in surgical decision-making, thereby reinforcing the meniscal repair’s role in achieving optimal patient outcomes and mitigating the progression of post-surgical osteoarthritis.

In the research conducted by Husen et al., [76] the benefits of meniscal repair over meniscectomy in patients aged 60 and older were markedly demonstrated, with the repair group showing significantly higher clinical outcomes (IKDC: 78.9 vs. 56.0; KOOS: 86.6 vs. 61.7; Lysholm: 88.3 vs. 68.7) compared to the meniscectomy group. Despite a clinical failure rate of 22% in the repair group, these results highlight the potential for improved quality of life and knee function through meniscal repair in the elderly population. This aligns with our review’s emphasis on meniscal preservation, extending its applicability to older patients, thereby challenging the notion that advanced age diminishes the viability of such reparative procedures.

In our systematic review, the selection of studies was heavily predicated on the assessment of osteoarthritis development, which inherently necessitates a substantially extended follow-up period for accurate evaluation, with an average of more than 50 months in each study group included in this review. This contrasts with the study by Lee et al. [77], where the median follow-up duration was 18 months, a timeframe that might not fully capture the long-term outcomes essential for observing osteoarthritis progression post-meniscus surgery. While the Lee et al. findings affirm the efficacy of both meniscectomy and meniscal repair in the short to medium term, showing significant improvements in the IKDC scores post-surgery, their observation of meniscal repair outcomes maintaining stability in late follow-up (beyond 18 months) aligns with our review’s emphasis on the durability of repair benefits. Specifically, Lee et al. noted an IKDC score improvement from 45.9 to 84.4 after repair, highlighting the sustained efficacy of this intervention. However, the dip in IKDC scores from 88.2 to 72.1 in meniscectomy patients during late follow-up (>18 months) underscores our systematic review’s concern over meniscectomy’s long-term implications. This juxtaposition underscores the necessity for prolonged follow-up in evaluating osteoarthritis development, further solidifying meniscal repair’s superiority in ensuring long-term knee health and functionality.

The clinical value of this systematic review is paramount for informing best practices in the treatment of posterior medial meniscus injuries, with a particular focus on the long-term outcome of osteoarthritis development. The aggregated evidence clearly indicates that meniscal repair is superior to meniscectomy in preventing the progression of OA. This is crucial, as OA is a leading cause of disability and pain, significantly impacting patients’ quality of life.

### 4.2. Limitations

This systematic review, while providing crucial insights into the impact of meniscectomy versus meniscal repair on osteoarthritis development, is not without limitations. First, the reliance on retrospective cohort studies introduces a risk of bias and limits the ability to establish causality. The variability in follow-up durations across studies may also affect the comparability of long-term outcomes, particularly the development of osteoarthritis. Additionally, the heterogeneity in patient demographics, such as age and baseline knee function, could influence the generalizability of the findings. Moreover, the study’s conclusions are drawn from aggregated outcome data without access to raw participant data or variance measures, precluding a comprehensive meta-analysis including heterogeneity assessment and weighted comparisons. This limitation emphasizes the need for interpretive caution and further research with more detailed data reporting. 

## 5. Conclusions

The systematic review conclusively demonstrates that meniscal repair should be the preferred surgical option over meniscectomy for patients with medial meniscus tears in the posterior root region, in order to minimize the risk of OA development. The evidence shows that meniscal repair provides superior long-term outcomes in terms of knee function, subjective patient assessments, and MRI findings, slowing the progression of osteoarthritis. Consequently, these findings should prompt clinical practice towards preserving the meniscus whenever feasible. The preservation of meniscal integrity appears to be a key factor in maintaining joint health and preventing OA, which is particularly important given the challenging nature of treating OA and its impact on patients’ lives. 

## Figures and Tables

**Table 1:** Study characteristics [51,52,53,54,55,56].

Study & Author	Country	Study Year	Study Design	Quality of Evidence
1 [51] Kim et al.	South Korea	2011	Retrospective cohort	Medium
2 [52] Chung et al.	South Korea	2015	Retrospective cohort	Medium
3 [53] Bernard et al.	United States	2019	Retrospective cohort	Medium
4 [54] Kim et al.	South Korea	2019	Retrospective cohort	Medium
5 [55] Dzidzishvili et al.	Spain	2022	Case-control	Medium
6 [56] Su et al.	China	2022	Retrospective cohort	Medium

**Table 2:** Patient characteristics [51,52,53,54,55,56].

Study Number	Sample Size	Age (Years)	Gender Distribution (Men)	BMI	Follow-Up (Months)
1 [51] Kim et al.	Meniscectomy: 28 Repair: 30	Meniscectomy: 57.4 Repair: 55.2	Meniscectomy: 14.3% Repair: 13.3%	Meniscectomy: 27.3 Repair: 26.8	Meniscectomy: 46.0 Repair: 48.5
2 [52] Chung et al.	Meniscectomy: 20 Repair: 37	Meniscectomy: 55.0 Repair: 55.5	Meniscectomy: 20% Repair: 10.8%	Meniscectomy: 27.4 Repair: 26.1	Meniscectomy: 67.5 Repair: 72.0
3 [53] Bernard et al.	Meniscectomy: 15 Repair: 15	Meniscectomy: 48.8 Repair: 46.1	Meniscectomy: 33.3% Repair: 33.3%	Meniscectomy: 33.9 Repair: 32.0	Meniscectomy: 66.2 Repair: 75.2
4 [54] Kim et al.	Meniscectomy: 24 Repair: 21	Meniscectomy: 55.9 Repair: 58.8	Meniscectomy: 87.5% Repair: 90.4%	Meniscectomy: 26.6 Repair: 25.9	Meniscectomy: 37.2 Repair: 39.2
5 [55] Dzidzishvili et al.	Meniscectomy: 35 Repair: 30	Meniscectomy: 56.0 Repair: 52.2	Meniscectomy: NR Repair: NR	Meniscectomy: 28.7 Repair: 28.5	Meniscectomy: 52.4 Repair: 27.2
6 [56] Su et al.	Meniscectomy: 22 Repair: 21	Meniscectomy: 57.7 Repair: 62.0	Meniscectomy: 9.1% Repair: 19.0%	Meniscectomy: 25.5 Repair: 26.2	Meniscectomy: 46.3 Repair: 42.4

NR—Not Reported; BMI—Body Mass Index.

**Table 3:** Disease characteristics and preoperative data [51,52,53,54,55,56].

Study Number	Kellgren–Lawrence	IKDC	Lysholm	Other Findings
1 [51] Kim et al.	Grade 3–4 Meniscectomy: 33.3% Repair: 25%	Meniscectomy: 42.3 Repair: 42.6	Meniscectomy: 56.0 Repair: 56.8	Joint space (mm) Meniscectomy: 6.1 Repair: 6.3
2 [52] Chung et al.	Grade 3–4 Meniscectomy: 0.0% Repair: 0.0%	Meniscectomy: 37.9 Repair: 40.1	Meniscectomy: 51.0 Repair: 52.3	Joint space (mm) Meniscectomy: 4.6 Repair: 4.8 Tegner score: Meniscectomy: 2.7 Repair: 2.7
3 [53] Bernard et al.	Median score Meniscectomy: 1.2 Repair: 1.6	Meniscectomy: NR Repair: NR	Meniscectomy: NR Repair: NR	Meniscectomy: NR Repair: NR
4 [54] Kim et al.	Grade 3–4 Meniscectomy: 0.0% Repair: 0.0%	Meniscectomy: 40.9 Repair: 39.7	Meniscectomy: 52.1 Repair: 51.7	Joint space (mm) Meniscectomy: 4.5 Repair: 4.7
5 [55] Dzidzishvili et al.	Grade 3–4 Meniscectomy: 34.3% Repair: 20.0%	Meniscectomy: NR Repair: NR	Meniscectomy: NR Repair: NR	Outerbridge grade 3–4 Meniscectomy: 54.3% Repair: 26.6% LaPrade grade 3–4 Meniscectomy: 77.2% Repair: 34.5%
6 [56] Su et al.	Median score Meniscectomy: 1.0 Repair: 1.0	Meniscectomy: 26.6 Repair: 24.1	Meniscectomy: 39.4 Repair: 38.7	Joint space (mm) Meniscectomy: 3.4 Repair: 3.2

NR—Not Reported; IKDC—International Knee Documentation Committee.

**Table 4:** Postoperative outcomes and conclusions [51,52,53,54,55,56].

Study Number	Kellgren–Lawrence	IKDC	Lysholm	Outcomes	Conclusions
1 [51] Kim et al.	Progression: Meniscectomy: 75.0% Repair: 30.0%	Meniscectomy: 74.1 ± 4.0 Repair: 77.2 ± 6.3	Meniscectomy: 81.6 ± 4.0 Repair: 85.1 ± 5.8	Complete healing (Repair): 56.7% Medial meniscal extrusion (Repair): decrease from 3.13 to 2.94 Arthrosis progression (Repair): 53.7% TKR: Meniscectomy: 10.7% Repair: 0.0%	Arthroscopic pullout repair for medial MRT yielded superior outcomes to partial meniscectomy, demonstrating effective meniscal healing.
2 [52] Chung et al.	Progression: Meniscectomy: 85.0% Repair: 20.0%	Meniscectomy: 49.3 ± 23.5 Repair: 73.7 ± 11.1	Meniscectomy: 62.8 ± 24.9 Repair: 84.3 ± 12.1	Joint space (mm) Meniscectomy: +2.3 Repair: +0.9 Tegner score: Meniscectomy: −0.3 Repair: +0.9	Refixation was more effective than partial meniscectomy in clinical and radiologic outcomes, with better survival rates over 5 years.
3 [53] Bernard et al.	Meniscectomy: 2.5 Repair: 1.7	Meniscectomy: 74 Repair: 72.3	Meniscectomy: NR Repair: NR	Tegner score: Meniscectomy: 4.3 Repair: 4.1 Progression to arthroplasty: Meniscectomy: 60.0% Repair: 0.0%	Meniscus root repair significantly reduces the progression to arthritis and need for knee arthroplasty, over nonoperative management and partial meniscectomy.
4 [54] Kim et al.	Progression Meniscectomy: 37.5% Repair: 38.1%	Meniscectomy: 71.5 ± 15.7 Repair: 75.2 ± 18.8	Meniscectomy: 75.9 ± 16.3 Repair: 80.9 ± 15.8	Joint space (mm) Meniscectomy: 3.5 Repair: 4.1 Progression to arthroplasty Meniscectomy: 16.7% Repair: 0.0%	The repair group had better functional and radiologic outcomes.
5 [55] Dzidzishvili et al.	Progression Meniscectomy: 57.1% Repair: 16.6%	Meniscectomy: 65.1 ± 18.2 Repair: 76.0 ± 13.3	Meniscectomy: 70 ± 20.5 Repair: 82 ± 14.2	Progression to arthroplasty Meniscectomy: 22.9% Repair: 10.0% Osteochondral defect Meniscectomy: 51.4% Repair: 23.3%	The repair had significantly improved clinical outcomes in middle-aged patients with mild knee osteoarthritis. Meniscal extrusion, osteochondral defect, and BMI > 30 were predictors of poor outcomes.
6 [56] Su et al.	Meniscectomy: NR Repair: NR	Meniscectomy: 71.3 ± 16.3 Repair: 73.7 ± 7.6	Meniscectomy: 82.5 ± 15.9 Repair: 86.6 ± 7.8	Joint space (mm) Meniscectomy: 5.0 Repair: 3.6 Complete healing (Repair): 71.4%	The repair group demonstrated less progression of articular cartilage wear and decreased meniscal extrusion, indicating better preservation of knee joint integrity.

NR—Not reported; TKR—Total knee replacement; IKDC—International Knee Documentation Committee.

## Data Availability

Not applicable.
